# Long-Term Transcriptomic Reprogramming in Peripheral Blood Mononuclear Cells of Severe COVID-19 Survivors Reveals Pro-Oncogenic Signatures and Cancer-Associated Hub Genes

**DOI:** 10.3390/v17121608

**Published:** 2025-12-12

**Authors:** Pelin Duru Cetinkaya, Ozgecan Kayalar, Vahap Eldem, Serap Argun Baris, Nurdan Kokturk, Selim Can Kuralay, Hadi Rajabi, Nur Konyalilar, Deniz Mortazavi, Seval Kubra Korkunc, Sinem Erkan, Gizem Tuse Aksoy, Gul Eyikudamaci, Pelin Pinar Deniz, Oya Baydar Toprak, Pinar Yildiz Gulhan, Gulseren Sagcan, Neslihan Kose Kabil, Aysegul Tomruk Erdem, Fusun Fakili, Onder Ozturk, Ilknur Basyigit, Hasim Boyaci, Emel Azak, Tansu Ulukavak Ciftci, Ipek Kivilcim Oguzulgen, Hasan Selcuk Ozger, Pinar Aysert Yildiz, Ismail Hanta, Ozlem Ataoglu, Merve Ercelik, Caglar Cuhadaroglu, Hacer Kuzu Okur, Muge Meltem Tor, Esra Nurlu Temel, Seval Kul, Yıldız Tutuncu, Oya Itil, Hasan Bayram

**Affiliations:** 1 Department of Pulmonary Medicine, Faculty of Medicine, Cukurova University, Adana 01790, Türkiye; 2 Department of Biology, Faculty of Arts and Sciences, Cukurova University, Adana 01790, Türkiye; 3 Koc University Research Center for Translational Medicine (KUTTAM), School of Medicine, Koc University, Istanbul 34010, Türkiye; 4 Department of Biology, Science Faculty, Istanbul University, Istanbul 34134, Türkiye; 5 Department of Pulmonary Medicine, Faculty of Medicine, Kocaeli University, Kocaeli 41380, Türkiye; 6 Department of Pulmonary Medicine, Faculty of Medicine, Gazi University, Ankara 06500, Türkiye; 7 Department of Pulmonary Medicine, Faculty of Medicine, Duzce University, Duzce 81620, Türkiye; 8 Department of Pulmonary Medicine, Altunizade Acibadem Hospital, Istanbul 34662, Türkiye; 9 Department of Pulmonary Medicine, Faculty of Medicine, Yalova University, Yalova 77200, Türkiye; 10 Department of Pulmonary Medicine, Faculty of Medicine, Zonguldak Bulent Ecevit University, Zonguldak 67100, Türkiye; 11 Department of Pulmonary Medicine, Faculty of Medicine, Gaziantep University, Gaziantep 27310, Türkiye; 12 Department of Pulmonary Medicine, Faculty of Medicine, Suleyman Demirel University, Isparta 32260, Türkiye; 13 Department of Infectious Disease and Clinical Microbiology, Faculty of Medicine, Kocaeli University, Kocaeli 41380, Türkiye; 14 Department of Infectious Diseases and Clinical Microbiology, Faculty of Medicine, Gazi University, Ankara 06500, Türkiye; 15 Department of Infectious Diseases and Clinical Microbiology, Faculty of Medicine, Suleyman Demirel University, Isparta 32260, Türkiye; 16 Department of Biostatistics, Faculty of Medicine, Gaziantep University, Gaziantep 27310, Türkiye; 17 Department of Immunology, Koc University Research Center for Translational Medicine (KUTTAM), School of Medicine, Koc University, Istanbul 34010, Türkiye; 18 Department of Pulmonary Medicine, Faculty of Medicine, Dokuz Eylul University, Izmir 35340, Türkiye; 19 Department of Pulmonary Medicine, School of Medicine, Koc University, Istanbul 34010, Türkiye

**Keywords:** COVID-19, transcriptomics, PBMCs, long-term effects, cancer risk, differentially expressed genes, bioinformatics analysis

## Abstract

This study examined the long-term transcriptomic reprogramming in peripheral blood mononuclear cells (PBMCs) of severe COVID-19 patients and its effects for cancer development. RNA sequencing was conducted on PBMCs obtained from healthy controls, COVID-19 patients without pneumonia, and COVID-19 patients exhibiting severe pneumonia one year post-infection. Differential gene expression analysis identified a sustained pro-oncogenic molecular signature, especially among severe COVID-19 patients. Functional enrichment analysis revealed a substantial enrichment of cancer-associated pathways, encompassing apoptosis, viral carcinogenesis, and transcriptional dysregulation. Notably, the autophagy-related gene SQSTM1/P62 was recognized as a distinctive hub gene within the severe COVID-19 patients, interacting with pivotal genes associated with inflammation, apoptosis, and cancer advancement. Survival analysis demonstrated that elevated expression of COVID-19-associated hub genes correlated with unfavorable prognosis in various cancer types, including adrenocortical carcinoma, bladder urothelial carcinoma, and brain lower-grade glioma. These findings indicate that severe COVID-19 infection may establish a systemic milieu favorable to cancer development or recurrence, highlighting the necessity of prolonged oncological monitoring in these patients. Finding specific molecular targets and pathways can help us understand how COVID-19 might be linked to a higher risk of cancer.

## 1. Introduction

It has been observed that symptoms may continue after the coronavirus disease 2019 (COVID-19) in the long term. Although various effects on the pulmonary, cardiovascular, musculoskeletal, and neuropsychiatric systems have been reported, there are many unknowns about their long-term effects [1,2,3]. These unknowns include cancer, whose access to diagnosis and treatment was restricted during the COVID-19 period. It is very important to determine the relationship between cancer and the long-term consequences of COVID-19 and its aftermath in the human body.

Studies have shown that the risk of complications of COVID-19 is higher in cancer patients [4,5,6,7]. A meta-analysis demonstrated that malignancy was associated with increased ICU admissions and mortality in COVID-19 patients [8], and Liu et al. (2021) reported that the risk of poor outcomes was approximately 2.3-fold higher in those with malignancy [9].

In a cohort from Guy’s Cancer Center, 51.3% of cancer patients who completed the survey developed long COVID, with fatigue, breathlessness, cognitive impairment, sleep disturbance, loss of taste, and depression being the most frequent symptoms. Breast, lung, and CNS cancer patients showed higher long-COVID rates than other cancer groups [10].

Concerns have grown regarding potential interactions between SARS-CoV-2 and cancer, especially given the rising global prevalence of malignancies. SARS-CoV-2 interacts with proteins involved in metabolism, DNA damage responses, cellular replication, and epigenetic regulation [11], and COVID-19–induced inflammation may influence tumor cells and their microenvironment [12,13]. A pivotal study showed that sera from COVID-19 patients promoted epithelial–mesenchymal transition in lung, breast, and colon cancer cell lines, increasing invasion and metastatic potential; furthermore, lung metastasis progressed in two patients six months after SARS-CoV-2 infection [14]. These findings suggest a link between inflammation and tumorigenesis.

The hidden pathophysiological mechanisms that support the close relationship between malignant tumors and the increased severity of COVID-19 disease are not fully understood. One possible explanation may be that patients with malignancies are more exposed to renin-angiotensin system (RAS) processes such as angiogenesis, cell proliferation, immune responses, and fibrosis [15]. On the other hand, in a study discussing whether SARS-CoV-2 may have an oncogenic potential and its capacity to cause cancer, it was emphasized that it is not yet known whether SARS-CoV-2 works by blocking tumor suppressor molecules such as P53 and stimulating the activation of oncogenes, similar to oncogenic viruses such as HPV, HBV, HBC, HHV4, EBV, HHV8, KSHV, MCPyV and HTLV-1. In another study, it was stated that SARS-CoV-1 and MERS-CoV viruses stimulated various pathways leading to carcinogenesis by suppressing the tumor suppressor retinoblastoma protein by non-structural protein (nsp)-15 [16]. Despite the oncogenic potential of SARS-CoV-1 and MERS-CoV, studies on the long-term symptoms of these viruses have not revealed any association between these viruses and cancer. In the study carried out by Chen et al., it was shown that SARS-CoV-2 proteins trigger the replication and lytic reactivation of a Kaposi’s sarcoma-associated herpesvirus (KSHV), which is considered one of the main viruses that cause cancer [17]. In another study, it was shown that in mice infected with SARS-CoV-2, the coronavirus helped stimulate metastatic breast cancer cells that were dormant. In another study, it has been shown in studies conducted independently of COVID-19 that neutrophil extracellular traps (NETs), which play an important role in the pathogenesis of COVID-19, may be a factor that triggers the exit of breast cancer cells from the dormant state [13,18]. In our previously published study, we showed that neutrophiles and NET formation signals are increased in the peripheral blood mononuclear cell (PBMC) transcriptome of severe COVID-19 patients [19]. As shown in these studies, data on whether SARS-CoV-2, which is an oncogenic virus and stimulates inflammation and inflammation-related carcinogenesis, has a cancer-stimulating effect in the short and long term is still insufficient. However, we believe that the present study will increase efforts to investigate the possibilities of severe COVID-19 stimulating cancer.

Although the mechanisms of the relationship between COVID-19 and cancer can be demonstrated theoretically and by looking at studies with other cancer-stimulating viruses, there is insufficient evidence associated with SARS-CoV-2-induced cancer cases. As a result of transcriptomic analysis of PBMCs of a total of 206 COVID-19 patients from three different datasets, COVID-19 was associated with cell cycle regulation and the regulation of cancer genes involved in cellular senescence processes. This study is related to the acute phase of COVID-19 and only yields bioinformatics results [20].

Whether severe COVID-19 induces long-term PBMC alterations capable of activating cancer-related pathways is still unknown. Identifying viral contributions to human cancer provides opportunities for prevention and treatment; however, no specific cancer cases have been robustly documented following COVID-19 infection aside from limited observations [14].

The aim of this study was to obtain data on whether the PBMC-related cellular and molecular infrastructure of cancer pathogenesis can be formed in these patients after long-term follow-up of COVID-19 disease.

## 2. Method

### 2.1. Study Design and Participants

This research involved patients from the previously published TURCOVID trial [21,22]. The methodology used in this study and our RNAseq dataset were presented in detail in our previous bioinformatics study, in which we presented our findings on the long-term transcriptomic consequences of severe COVID-19 [19]. Between 11 March and 18 July 2020, during the initial period of the COVID-19 pandemic, 1500 patients aged 18 and older, who were monitored and treated for COVID-19, were incorporated into the targeted trial population of the multi-center TTS-TURCOVID-19 registry cohort. A total of 831 patients were included in the trial at 13 of the 26 locations (11 university hospitals, 2 significant tertiary institutions, and 1 private hospital). A standardized questionnaire was administered to these patients in the cohort via phone calls following the acquisition of signed informed consent. Out of the cohort of 831 patients, 230 (27.7%) were unreachable, 28 (3.4%) declined participation, 69 (8.3%) were eliminated owing to mortality, leaving 504 (60.6%) patients included in the study [21]. One year after the initial evaluation, 504 patients were contacted via telephone and invited for follow-up. A total of 138 patients from 11 participating centers who consented to participate and attended the follow-up visit provided written informed consent, after which comprehensive clinical, laboratory, and radiological assessments were conducted.

A comprehensive assessment (clinical, laboratory, and radiological) of the patients was conducted. Among the established groups, one had 13 patients (eight male; five female) who exhibited no radiological pneumonia upon detection of COVID-19 infection, while the other group included 14 cases (10 male; 4 female) that presented with clinically and radiologically severe pneumonia. The control group consisted of 13 participants (eight males and five females) who remained uninfected. Age, gender, and smoking status were considered to mitigate confounding variables during the randomization process. PBMCs were isolated from patient blood samples and preserved at −80 °C until RNA extraction was performed. After evaluating RNA quality, samples from two patients without pneumonia and four patients with severe pneumonia were eliminated from the study due to poor RNA quality.

Accordingly, the RNA and overall quality analysis of PBMC samples revealed the following case distribution among the three study groups: (i) COVID-19 patients without pneumonia (no pneumonia, NP; n = 11), (ii) COVID-19 patients with severe pneumonia (severe pneumonia, SP; n = 10), and (iii) healthy subjects without SARS-CoV-2 infection (healthy controls, C; n = 13). The comprehensive demographic data of the study were presented in our prior research [19]. In the analysis of long-term clinical symptoms of COVID-19 among patient groups, 4 out of 11 patients without pneumonia exhibited long-term symptoms, while 5 out of 10 patients with severe pneumonia also displayed long-term symptoms, with no significant difference observed between the two groups.

### 2.2. Blood Sample Collection and Isolation of Peripheral Blood Mononuclear Cells (PBMCs)

From each participant, almost 20 mL of venous blood was obtained. Peripheral blood mononuclear cells (PBMC) were isolated from the collected blood using a benchtop centrifuge utilizing Lymphoprep™ (Alere Technologies, Oslo, Norway) solution at 2000 rpm for 20 min.

### 2.3. Total RNA Isolation, Purity, Quantity, and Integrity Analysis from PBMCs

Total RNA was extracted from the isolated PBMCs utilizing Trizol (Thermo, Carlsbad, CA, USA) in accordance with the manufacturer’s guidelines, and the resultant RNAs were purified employing the RNA isolation kit (Zymo, Irvine, CA, USA). The extracted RNAs were quantified using the A260/280 technique on the Nanodrop 2000c instrument (Thermo Fisher Scientific, Waltham, MA, USA), with values between 1.8 and 2.2 deemed pure. The integrity of the extracted RNAs was assessed utilizing the RNA Nano 6000 test kit (Agilent Technologies, Santa Clara, CA, USA) within the Agilent Bioanalyzer 2100 System. A library was constructed from samples exhibiting an RNA integrity value (RIN) of 7.5 or higher.

### 2.4. Transcriptome Library Preparation and RNA Sequencing

Before library preparation, mRNAs were enhanced by eliminating ribosomal RNA (rRNA) with the MGIEasy rRNA Depletion Kit (MGI Tech, Shenzhen, Guandong, China). Subsequently, total elimination of DNA was accomplished with the use of DNase I (NEB). RNA libraries were generated from 500 ng of RNA using the MGIEasy RNA Library Prep Kit V3.0 (MGI, Shenzhen, China) in accordance with the manufacturer’s guidelines. Initially, enriched RNA samples were subjected to fragmentation in a buffer solution. Subsequently, the second chain was produced using the generated short fragments and reverse transcription enzymes.

The acquired cDNA fragments underwent normal library preparation procedures, including end repair, poly A tail addition, and adapter ligation. Following the purification process utilizing DNA cleansing beads, the adapter-ligated fragments underwent enrichment through 14 PCR cycles, were denatured, and subsequently subjected to a single-chain circularization reaction to generate a single-strand circular DNA library. The libraries were subsequently utilized to generate DNA nanoballs (DNBs) that facilitate circular replication (RCR). The acquired DNBs were subsequently introduced into preformed patterned nanoarrays, and the sequencing procedure was conducted using the DNBSEQ-G400 sequencing apparatus with a paired-end length of 100 bp.

### 2.5. Analysis of RNA Sequencing Data and Differential Gene Expression Analysis

**Figure 1:**
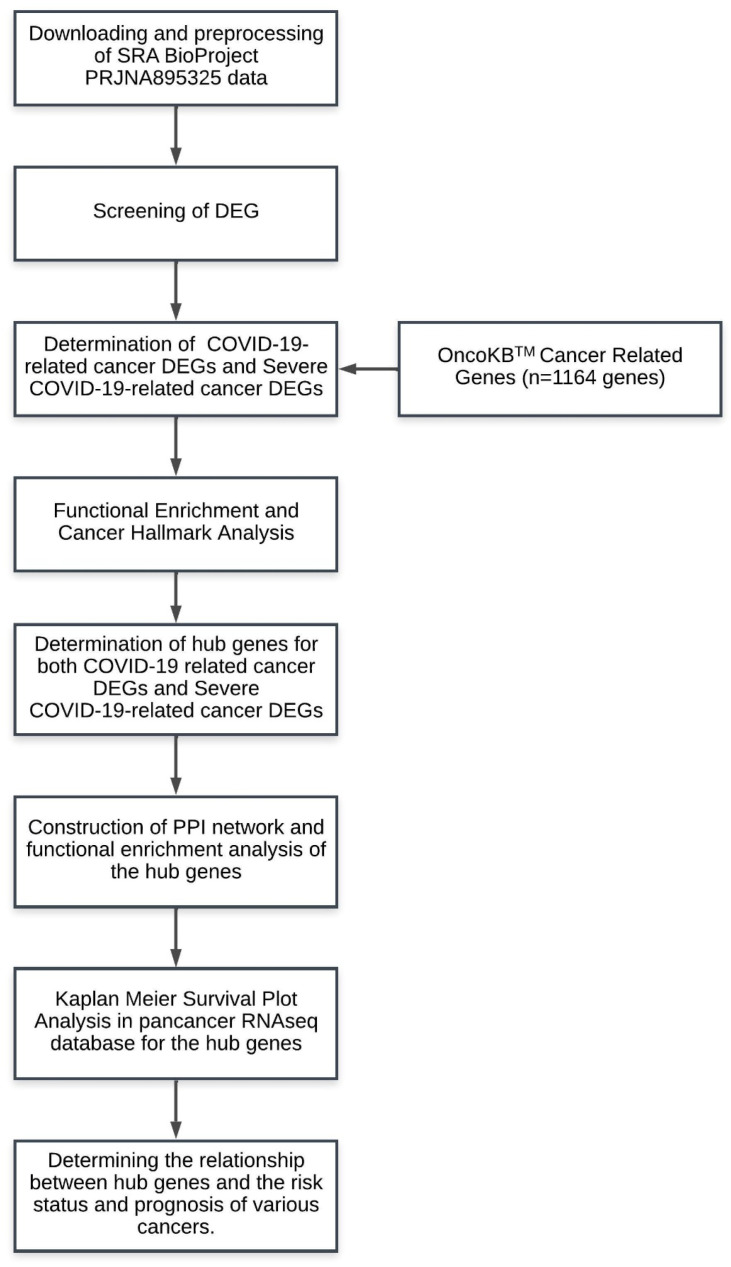
Flowchart of the research.

The quality of raw sequencing reads was assessed using FastQC (Babraham Bioinformatics, Cambridge, UK) prior to and subsequent to sequence trimming. MultiQC software v1.19 was utilized to consolidate the outcomes of FastQC for a comparative analysis of the characteristics of all RNA-Seq libraries [23]. Raw readings were processed with fastp v0.23.0 to exclude adaptor contamination, ambiguous bases (N > 5), low-quality reads (Phred score, Q < 20), and fragments less than 30 nt [24]. All alternative selections employed the default settings. Summary statistics for RNA-Seq readings were generated utilizing seqkit v2.0.0 [25]. Filtered reads are aligned to the human reference genome (GRCh38.p13, Ensembl Release 106) with Hisat2 v2.2.1 [26]. The alignment statistics were obtained with Sambamba v0.8.0 [27]. Count matrices and gene-level assignments were produced using featureCounts from the Subread software v2.0.0 [28] with annotation version GRCh38.106 (Ensembl “.gtf”). Differential gene expression analysis between groups was conducted on raw counts with DESeq2 v1.34.0 following variance-stabilizing transformation (vst) normalization [29]. Genes were deemed substantially differentially expressed if the adjusted *p*-value was below 0.001 and log2FC above 1.0, utilizing the Benjamini–Hochberg (BH) multiple test correction method. The intersection between DEGs in three pairwise comparisons was demonstrated via a Venn diagram web tool (https://molbiotools.com/listcompare.php/ accessed on 21 November 2024) (Figure 1).

### 2.6. Functional Annotation and Enrichment Analysis

Functional enrichment analysis of DEG among all groups, including control (C), no pneumonia (NP), and severe pneumonia (SP), was performed with NetworkAnalyst and visualized with Ridgeline graphics [30]. The functional analyses of common and hub genes were performed using STRINGdb (version 12.0), using Homo sapiens (Ensembl Release 104) as background for enrichment [31]. The significance of enrichment analysis was estimated by Benjamini–Hochberg false discovery rate (FDR) < 0.05 correction. Protein–protein interaction (PPI) network analyses were conducted utilizing STRINGdb, with a minimum necessary interaction score of medium confidence (0.400). Analysis was conducted based on interaction evidence ratings. Texminnig, experiments, databases, co-expression, neighborhood, gene fusion, and co-occurrence were identified as active interaction sources. STRINGdb’s KEGG, GO (biological processes, molecular function, and cellular components), and disease–gene interaction visualization capabilities were employed for functional enrichment analysis [31] (Figure 1).

### 2.7. Determination of Cancer-Related DEGs Related to Long-Term COVID-19 and Long-Term Severe COVID-19 and Their Cancer Hallmarks Pathways

A list of 1164 genes consisting of oncogene and tumor suppressor genes was downloaded from the OnkoKB^TM^ database (https://www.oncokb.org/cancer-genes/ accessed on 23 November 2024) to compare the common genes associated with severe COVID-19 obtained as a result of pairwise comparisons, and this gene list was compared with common DEGs in our study [32,33] (Figure 1). Thus, cancer-related genes in our gene set, whose expression changed significantly, were identified. Using the Cancer Hallmark web tool [34], cancer gene signatures and associated cellular pathways were examined in long-term transcriptomic changes in patients with blood collection during the one-year follow-up period after COVID-19 and severe COVID-19.

### 2.8. Determination of Cancer-Related-Hub Genes

We utilized the cytoHubba (version 0.1) plug-in of Cytoscape (version 3.10.2, accessed on 25 November 2024) to evaluate the hub genes. We identified twelve frequently utilized algorithms (MCC, DMNC, MNC, Degree, EPC, BottleNeck, EcCentricity, Closeness, Radiality, Betweenness, Stress, and Clustering Coefficient) via the plug-in to evaluate and choose cancer-related hub genes for both COVID-19 and severe COVID-19 (Figure 1). We visualized this analysis using the Upset plot subfunction of Chiplot, a free online tool (https://www.chiplot.online/upset_plot.html accessed on 25 November 2024). Subsequently, we identified the hub genes. Subsequently, we constructed a protein–protein interaction network of these hub genes utilizing STRING PPI [31], a dependable instrument for discerning internal relationships among gene sets. Our RNAseq investigation of both COVID-19 and Severe COVID-19 hub genes revealed differential expression among the groups. Furthermore, KEGG pathway analysis was conducted to examine the pathways in which the network linked to the hub gene discovered for severe COVID-19 was involved.

### 2.9. Gene Signature Analysis and Kaplan–Meier Plotter

First of all, we analyzed cancer-related gene signatures in 21 solid tumor types with the gene outcome module of TIMER2.0 (Figure 1). The module enables users to swiftly assess the correlation between immune subgroup abundance and patient survival across TCGA cancer types. TIMER2.0 assesses the impact of specific gene expression and the degree of immune infiltration on patient clinical outcome using a Cox proportional hazard model [35].

The Kaplan–Meier plotter (http://kmplot.com/analysis/ accessed on 28 November 2024) is utilized to assess the correlation between the expression of various genes (mRNAs, miRNAs, proteins) and survival across 21 solid tumor types, including bladder cancer, liver cancer, lung cancer, and gastric carcinoma [36]. The data sources are the Gene Expression Omnibus (GEO) and the Cancer Genome Atlas (TCGA). We utilized the Pan-cancer RNA-seq component of the KMplot website to examine the survival of prevalent pivot genes. This investigation evaluated the risk value (adjusted *p*-value < 0.05) of various prevalent pivot genes, all of which were statistically significant. The evaluation assessed the impact of low and high gene expression on survival in the tumor group relative to the control group.

## 3. Results

### 3.1. Identification of DEGs Among Healthy Controls, Post-COVID-19 Patients with No Pneumonia, and Post-COVID-19 Patients with Severe Pneumonia and the Functional Enrichment Analysis of the Pairwise Comparisons

Firstly, we analyzed our raw data from the Bioproject number PRJNA895325, which we previously published in the NCBI short read archive. With this analysis, RNA sequencing data of 34 RNA samples obtained from healthy individuals who did not have COVID-19 (Control Group, CNT, n = 13), the post-COVID-19 NP group (n = 11), and the post-COVID-19 SP group (n = 10) were analyzed to obtain a list of differentially expressed genes (DEGs).

The term “CNT vs. NP” denotes genes that exhibit up-regulation or down-regulation in the NP group in comparison to the control group. “C vs. SP” denotes genes that exhibit upregulation or downregulation in the SP group relative to the control. The “NP vs. SP” group denotes the genes that exhibit upregulation or downregulation in the SP group relative to the NP group.

**Figure 2:**
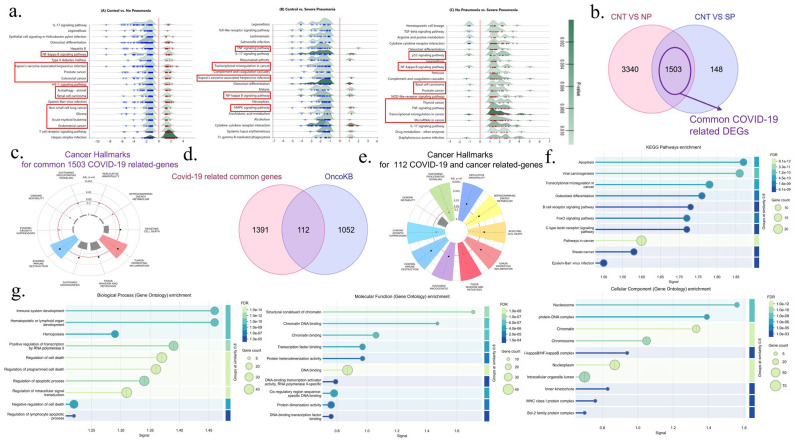
COVID-19-related common genes and their relations to cancer pathways. (**a**) Ridge plots show the functional enrichment analysis of DEGs in the pairwise comparisons include CNT vs. NP, CNT vs. SP, and NP vs. SP. (**b**) Venn diagram shows pairwise comparisons including CNT vs. NP (red), CNT vs. SP (purple) and NP vs. SP (green), and 1503 COVID-19 related common genes, (**c**) Cancer hallmarks enrichment of 1503 COVID-19 related common genes, (**d**) Venn diagram shows the comparison of these common genes (red) with cancer-associated genes in the OncoKB^TM^ database (purple). (**e**) Cancer hallmarks enrichment of 112 common cancer-related COVID-19 genes. (**f**) KEGG analysis of 112 common genes. (**g**) The figure shows GO (BP, MF, and CC) enrichment analysis of these genes. In bubble plots, the lengths of the horizontal rods indicate the strength of the signal, the colors indicate statistical significance, and the width of the round shape at the ends indicates the magnitude of the number of genes involved.

We found 4843 DEGs in the comparison of C and NP, comprising 1839 downregulated (down) and 3004 upregulated (up) genes. We identified 1651 differentially expressed genes (DEGs), comprising 1566 upregulated and 85 downregulated genes, in the comparison between C and SP (C vs. SP). In the comparison between NP and SP, there are 954 differentially expressed genes (DEGs), comprising 79 upregulated and 875 downregulated genes. According to the statistical cut-off values determined in the methodology, the general functional pathway analysis (KEGG) of DEGs obtained after pairwise comparisons of “C vs. NP”, “C vs. SP” and “NP and SP” showed that cancer-related signaling pathways were enriched in PBMC transcriptomics 1 year later in patients who had COVID-19 without pneumonia and those who had severe pneumonia compared to the healthy group without COVID-19. In the C vs. NP comparison, cancer signaling pathways such as NFKB, HIF1, and apoptosis signaling pathways, and prostate cancer, colorectal cancer, endometrial cancer, non-small cell lung cancer, renal cell carcinoma, glioma, and acute myeloid leukemia were significantly enriched. On the other hand, herpes sipmlex, Epstein–Barr, and Kaposi sarcoma-associated herpesvirus infection signaling pathways were also enriched in this comparison. In the C vs. SP comparison, it was determined that TNF, NFKB, and MAPK signaling pathways, along with transcriptional misregulation in cancer and Kaposi sarcoma-associated Epstein–Barr virus infection pathways, were significantly changed cancer-related pathways. In the NP vs. SP comparison, it was determined that p53, NFKB, and TNF signaling pathways, along with basal cell carcinoma, prostate cancer, thyroid cancer, transcriptional misregulation in cancer, and microRNA in cancer pathways were significantly changed cancer pathways in patients who had severe pneumonia compared to the group who did not have pneumonia during the period they had COVID-19 (Figure 2a).

### 3.2. Identification of COVID-19 and Severe COVID-19 Related-DEGs and Their Cancer Hallmarks

**Figure 3:**
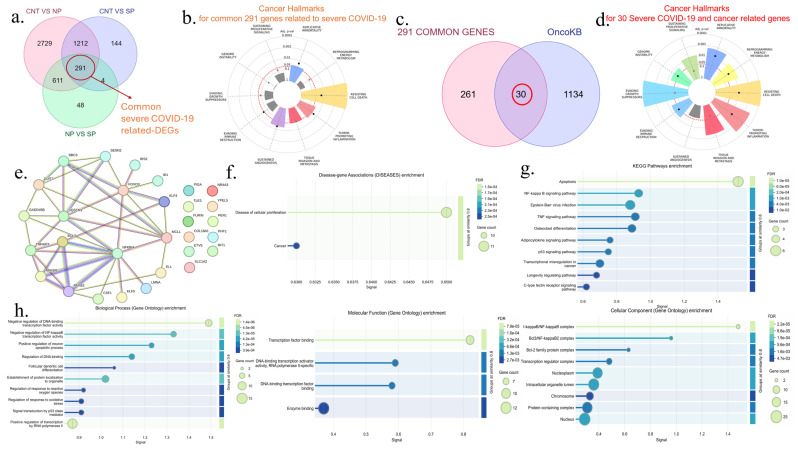
The analysis of 291 common severe COVID-19-related DEGs among healthy controls, no pneumonia, and severe pneumonia. (**a**) Venn diagram shows pairwise comparisons including CNT vs. NP (red), CNT vs. SP (purple) and NP vs. SP (green), and 291 severe COVID-19-related common genes, (**b**) Cancer hallmarks enrichment of 291 severe COVID-19 related common genes, (**c**) Venn diagram shows the comparison of these common genes (red) with cancer-associated genes in the OncoKB^TM^ database (purple). (**d**) Cancer hallmarks enrichment of 30 common cancer-related severe COVID-19 genes. (**e**) Protein–protein interaction network of these genes. (**f**) Diseases-gene interaction enrichment analysis of these genes. (**g**) KEGG analysis of 30 common genes. (**h**) The figure shows GO (BP, MF, and CC) enrichment analysis of these genes. In bubble plots, the lengths of the horizontal rods indicate the strength of the signal, the colors indicate statistical significance, and the width of the round shape at the ends indicates the magnitude of the number of genes involved.

In this study, we first identified COVID-19-related DEGs with “C vs. NP” and “C vs. SP” pairwise comparisons and analyzed whether they were in cancer-related genes. We identified 1503 common COVID-19-related DEGs (Figure 2b). Then, “C vs. NP”, “C vs. SP”, and “NP vs. SP” pairwise comparisons were compared to determine severe COVID-19-related genes. Totally, 291 severe COVID-19-related DEGs were identified (Figure 3a). After these genes were identified, it was determined whether they were cancer-related genes. Cancer Hallmark analysis of 1503 common COVID-19-associated genes revealed significant enrichment of tumor-promoting inflammation (adj *p*-val < 0.01) and evading immune destruction (adj *p*-val < 0.01) cancer hallmarks (Figure 2c). When 291 severe COVID-19-related genes were analyzed, it was determined that the most important cancer hallmark was resisting cell death (adj *p*-val < 0.0001), followed by replicative immortality (adj *p*-val < 0.05), tumor-promoting inflammation (adj *p*-val < 0.05), sustained angiogenesis (adj *p*-val < 0.05), and tissue invasion and metastasis (adj *p*-val < 0.05) hallmarks (Figure 3b).

### 3.3. Identification of Cancer-Related COVID-19 DEGs and Cancer-Related Severe COVID-19 DEGs and Their Functional Enrichment Analysis

The lists of 1503 common genes associated with COVID-19 alone and 291 genes associated with severe COVID-19 were compared with the OncoKB’s updated list of 1164 genes, including oncogenes, tumor suppressors, and other cancer-associated genes. Comparisons were shown with a Venn diagram. As a result of the comparisons, 112 overlapping COVID-19-associated cancer genes were identified (Figure 2d). In a comparison to identify severe COVID-19-specific cancer genes, 30 genes out of 291 genes were found to be associated with Severe-COVID-19-associated cancer (Figure 3c). Functional analysis of 112 COVID-19-associated cancer genes was performed by KEGG and GO. According to KEGG analyses, cancer-related signaling pathways such as apoptosis, viral carcinogenesis, transcriptional misregulation in cancer, pathways in cancer, and breast cancer were significantly enriched. According to the GO Biological process analysis, it was determined that processes such as positive regulation of transcriptional processes and negative regulation of cell death carried out by RNA polymerase II were significantly enriched. According to the GO Molecular function analysis, it was observed that genes associated with the binding of transcription factors to DNA and protein dimerization activities were enriched. According to Go cellular component analysis, it was determined that 112 genes are composed of proteins that interact with the intracellular organelle lumen, nucleoplasm, and chromosome (Figure 2f,g).

Protein–protein interaction analysis, enrichment of disease-gene relationships, and functional enrichment analyses of 30 cancer genes associated with severe COVID-19 were performed. According to the protein–protein interaction analysis, the interaction network of 19 genes was determined within 30 genes. The remaining 11 genes, including *PIGA*, *NR4A3*, *TLE3*, *YPEL5*, *FURIN*, *PER1*, *COL18A1*, *PHF1*, *ETV5*, *RIT1*, and *SLC1A2*, were not involved in this interaction. The enrichment *p*-value of this interaction is 6.1 × 10^−14^. (Figure 3e). According to the disease enrichment analysis, 30 genes were found to be composed of genes associated with cell proliferation disease and cancer (Figure 3f). According to KEGG’s analysis of these genes, apoptosis was determined to be the most important enriched cellular pathway. Other enriched KEGG pathways were NFKB, TNF, P53, and longevity-regulating signaling pathways. According to the GO Biological process analysis, the biological processes, including negative regulation of transcription factor activity, negative regulation of oxidative stress, and p53 signal regulation of NFKB binding to DNA, were significantly enriched. According to the GO Molecular Function analysis, it was determined that the binding and enzyme binding functions of transcription factors to DNA were enriched. Finally, according to the GO Cellular Component analysis of 30 vessels of severe COVID-19-associated cancer, it was determined that the I-kappaB/NF-kappaB complex, Bcl3/NF-kappaB2 complex, and Bcl2 family protein complexes in the nucleus were enriched (Figure 3g,h).

### 3.4. Cancer Hallmarks Analysis of COVID-19 Related Cancer DEGs and Severe COVID-19 Related Cancer DEGs

According to the cancer hallmark analysis of 112 COVID-19 related cancer DEGs, apart from genome instability from eight cancer hallmark feature signatures, seven hallmarks, including sustaining proliferative signaling, replicative immortality, reprograming energy metabolism, resisting cell death, tumor-promoting inflammation, tissue invasion and metastasis, sustained angiogenesis, evading destruction, and evading growth suppressors, were significantly prominent (Figure 2e). According to the cancer hallmark analysis of 30 severe COVID-19-related cancer DEGs, resisting cell death, evading growth suppressor, tumor-promoting inflammation, and replicative immortality were the most significant associations. These hallmarks were followed by significantly varying hallmarks of sustaining proliferative signaling, reprogramming energy metabolism, genome instability, and evading immune destruction. It has also been determined that these genes are not associated with the sustained angiogenesis hallmark (Figure 3d).

### 3.5. Selection and Analysis of Hub Genes, and Their Protein–Protein Interactions

**Figure 4:**
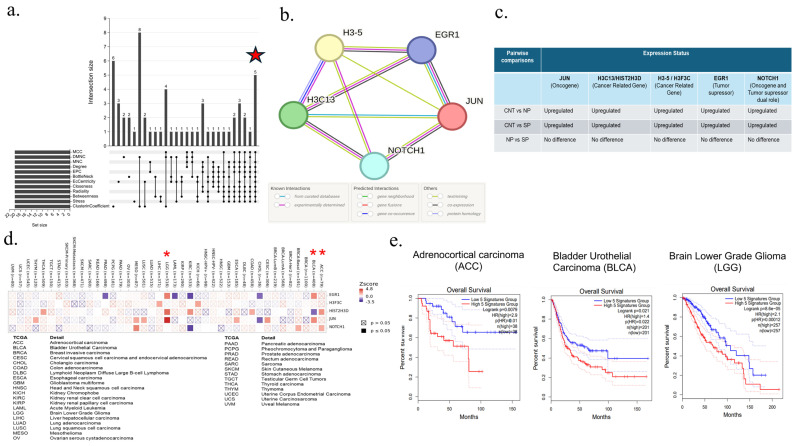
Upset diagram and protein–protein interaction network of COVID-19-related cancer hub genes. (**a**) Upset diagram showing the twelve algorithms screened for five overlapping central genes. (**b**) Hub genes and their interaction were visualized via STRINGdB. (**c**) Expression states of hub genes in CNT vs. NP, CNT vs. SP, and NP vs. SP pairwise comparisons (**d**) TIMER2.0 gene outcome analysis of hub genes (**e**) Kaplan–Meier survival plotter analysis of cancer types in which the expression of five hub genes is significantly increased.

**Figure 5:**
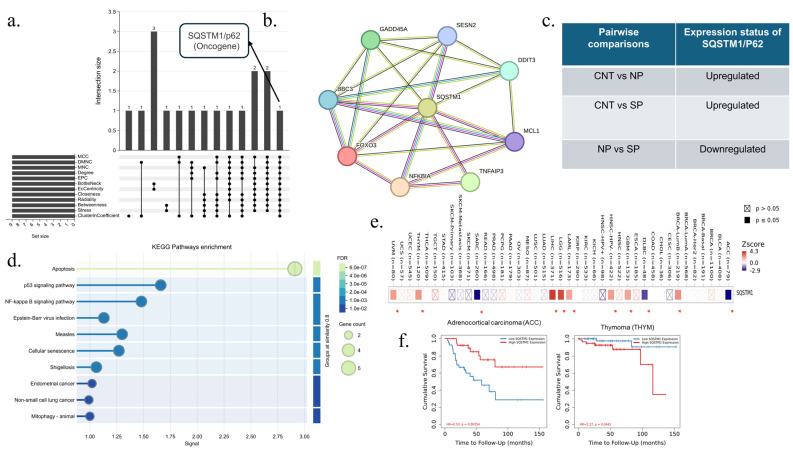
Upset diagram and protein–protein interaction network of severe-COVID-19 related cancer hub genes. (**a**) Upset diagram showing the twelve algorithms screened for one overlapping central gene. (**b**) *SQSTM1/P62* and its interaction were visualized via STRINGdB. (**c**) Expression states of the SQSTM1/P62 gene in CNT vs. NP, CNT vs. SP, and NP vs. SP pairwise comparisons (**d**) TIMER2.0 gene outcome analysis of the SQSTM1/P62 gene. (**e**) Kaplan–Meier survival plotter analysis of cancer types in which the expression of the gene is significantly upregulated or downregulated. (**f**) Kaplan-Meier plots illustrate the survival effects of low and high expression of the SQSTM1/P62 gene in adrenocortical carcinoma and thymoma. The blue line shows the survival level when the expression of SQSTM1 gene is low. The red line shows the survival level when the expression of this gene is high.

Using Cytoscape’s cytoHubba plug-in, the top 20 hub genes were sequenced using 12 algorithms: MCC, DMNC, MNC, Degree, EPC, BottleNeck, EcCentricity, Closeness, Radiality, Betweenness, Stress, and ClusterinCoefficient from 112 COVID-19-associated cancer genes. After the list of top 20 genes from each algorithm was intersected with the help of an upset diagram, 5 common central genes, including *H3-5*, *H3C13*, *EGR1*, *JUN*, and *NOTCH1*, were determined (Figure 4a). On the basis of the string protein–protein interaction network, the interaction of *JUN*,*H3-5*, and *H3C13* genes is noteworthy among these genes (Figure 4b). The expression levels of these five COVID-19-associated cancer hub genes were examined in pairwise comparisons of “CNT vs. NP”, “CNT vs. SP”, and “NP vs. SP”. All of these genes were upregulated in the “CNT vs. NP” and “CNT vs. SP” comparisons but did not change significantly in the NP vs. SP comparison (Figure 4c). Using the same program, the top 10 hub genes were sequenced using 12 algorithms out of 30 severe COVID-19-associated cancer genes. After intersecting the list of top 10 genes from each algorithm with the help of an upset diagram, it was determined that the *SQSTM1/P62* gene is a common central gene for severe COVID-19-associated cancer (Figure 5a). Out of 30 genes, this gene interacts only with *GADD45A*, *SESN2*, *MCL1*, *BBC3*, *FOXO3*, *NFKBIA*, *TNFAIP3*, and *DDIT3* (Figure 5b). The expression levels of the *SQSTM1/P62* gene were examined in pairwise comparisons of “CNT vs. NP”, “CNT vs. SP”, and “NP vs. SP”. This gene was upregulated in the “CNT vs. NP” and “CNT vs. SP” comparisons and downregulated in the NP vs. SP comparison (Figure 5c). KEGG analysis of this gene and its associated genes showed significant connectivity of apoptosis, p53, NFKB, and cellular senescence signaling pathways in this interaction network (Figure 5d).

### 3.6. Determination of Cancer Types That Are Risky Due to Hub Genes and Their Changes Using the Cumulative Survival Plotter

The relationship of five central genes associated with COVID-19 with 21 TCGA-based solid tumors was examined by TIMER2.0 Gene Outcome analysis. Further survival analysis was performed for adrenocortical carcinoma (ACC), bladder urothelial carcinoma (BLCA), and brain lower-grade glioma (LGG), in which two or more of these five genes were increased in patients with COVID-19 compared to patients who had not had COVID-19 (Figure 4d). While high expression of five genes does not pose a significant risk for other cancer types and mesothelioma, high levels of expression of these five COVID-19-associated hub genes are significantly associated with these cancer types and their poor prognosis for ACC (hazard ratio “HR” = 2.9, *p* = 0.01), BLCA (HR = 1.4, *p* = 0.022) and LGG (HR = 2.1, *p* = 0.00012) (Figure 4e). According to the analysis of the *SQSTM1/P62* gene, which is the hub gene associated with severe COVID-19, further survival analysis was performed for adrenocortical carcinoma (ACC), breast invasive carcinoma (BRCA), lymphoid neoplasm diffuse large B-cell lymphoma (DLBC), glioblastoma (GBM), HPV-negative head and neck squamous cell carcinoma (HNSC), acute myeloid leukemia (LAML), brain lower grade glioma (LGG), liver hepatocellular carcinoma (LIHC), sarcoma (SARC), thymoma (THYM), and uveal melanoma (UVM) (Figure 5e). Among these cancer types, it was determined that the increased expression of the *SQSTM1/P62* gene was associated with THYM (HR = 2.27, *p* = 0.0442) and its poor prognosis. On the other hand, decreased expression of this gene in patients with severe COVID-19 is also associated with ACC (HR = 0.53, *p* = 0.00254) and its poor prognosis (Figure 5f).

**Figure 6:**
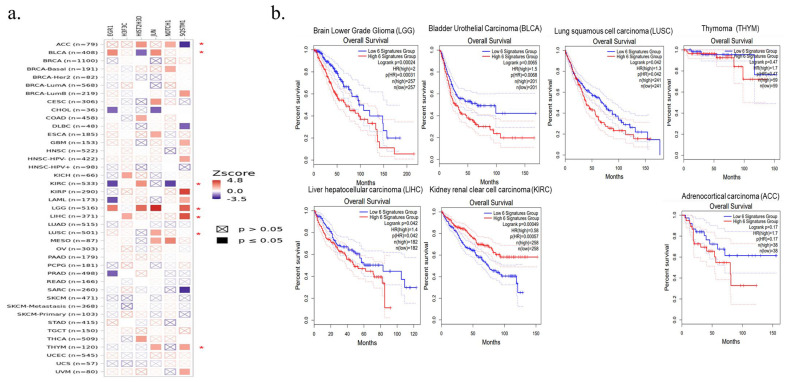
Cumulative cancer risk association of hub genes associated with COVID-19 and severe COVID-19. (**a**) Gene outcome analysis of *H3-5/H3F3C*, *H3C13/HIST2H3D*, *JUN*, *EGR1*, *NOTCH1* and *SQSTM1/P62*. (**b**) Kaplan–Meier survival plotter analysis of cancer types in which the expression of all six hub genes is significantly upregulated.

The increase in the expression of *SQSTM1* in patients who had COVID-19, together with five central genes associated with COVID-19. In the analysis we performed to determine all six genes together pose a risk for which cancer type and its prognosis, it was determined that the increase in the expression of these six hub genes worsened LGG (HR = 2, *p* = 0.00031) and BLCA (HR = 1.5, *p* = 0.0068) cancers and their prognosis. By including the increase in expression of the *SQSTM1* gene in the analysis of five genes, lung squamous cell carcinoma (LUSC; HR = 1.3, *p* = 0.042) and liver hepatocellular carcinoma (LIHC; HR = 1.4, *p* = 0.042). Conversely, adrenocortical carcinoma (ACC; HR = 1.7, *p* = 0.17) and renal clear cell carcinoma (KIRC; HR = 0.58, *p* = 0.0057) and their poor prognosis. An increase in *SQSTM1/P62* and five other hub genes was found to reduce the risk and prognosis of THYM (HR = 1.7, *p* = 0.47) (Figure 6a, b).

## 4. Discussion

This study presents the first evidence of a persistent pro-oncogenic molecular signature at the transcriptomic level, continuing even one year after infection, particularly in individuals who experienced severe COVID-19. This sustained reprogramming, which we observed in PBMCs, strengthens the hypothesis that COVID-19 is not merely an acute respiratory illness but may also prime a systemic environment for cancer development or recurrence. Our findings demonstrated an enrichment of pathways associated with the hallmarks of cancer—specifically “evading cell death,” “sustaining proliferative signaling,” and “tissue invasion and metastasis”—in the PBMCs of severe COVID-19 survivors. This indicates the formation of a systemic microenvironment that supports tumorigenesis or metastatic reactivation throughout the body. Notably, our identification of H3-5/H3F3C, H3C13/HIST2H3D, JUN, EGR1, NOTCH1, and SQSTM1/P62 as central hub genes unique to the COVID-19 signature links this pro-oncogenic state to a specific molecular target that regulates inflammation, autophagy, and cancer progression.

The central finding of our work—that COVID-19 creates a persistent pro-inflammatory and immunosuppressive state—is now strongly supported by powerful new evidence in the literature. A recent groundbreaking study published in Nature directly proved this hypothesis using both mouse models and human data. Chia et al. (2024) showed that both influenza and SARS-CoV-2 infections woke up dormant breast cancer cells in the lungs within days, causing them to develop into large metastatic lesions in as little as two weeks [37]. This process was found to be dependent on the cytokine Interleukin-6 (IL-6), which plays a central role in COVID-19 pathogenesis. Crucially, these experimental findings were validated by observational data from large human databases like UK Biobank and Flatiron Health. These analyses revealed a significantly increased risk of cancer-related death and lung metastasis in cancer patients who contracted SARS-CoV-2 compared to those who were not infected [37]. Similarly, another study by Qian et al. (2025) showed that respiratory viral infections epigenetically reprogram the lung microenvironment to create an “inflammatory memory,” which accelerates subsequent tumor growth [38]. Our study extends these findings by demonstrating that this effect is not confined to a local site but is systemic via PBMCs and persists for up to one year after infection, shedding light on the lasting systemic immunological mechanism underlying the findings of the previous studies.

The potential mechanisms underpinning COVID-19’s ability to increase cancer risk are manifold. The increase in the “tissue invasion and metastasis” hallmark identified in our study aligns with the findings of Saygideger et al. (2021), who showed that serum from COVID-19 patients triggers EMT in cancer cells and increases the expression of metastasis-related genes [14]. This confirms that systemic factors circulating during COVID-19 can directly enhance the motility and invasive capacity of cancer cells. As shown by Chia et al. (2024), one of the most critical of these systemic factors is IL-6, which triggers the awakening of dormant cancer cells [37]. Our identification of NOTCH1 and SQSTM1/P62 as key enriched genes provides mechanistic support for this finding. NOTCH1 signaling is critically involved in cell fate and oncogene-induced senescence and differentiation, and its abnormalities are linked to Epithelial–Mesenchymal Transition (EMT) and metastatic invasion, supporting the observed “tissue invasion and metastasis” feature [39,40,41,42,43,44,45,46]. Furthermore, SQSTM1/P62, an autophagic regulator, has been shown to play a role in regulating the COVID-19-induced inflammatory response and can stimulate cancer cell migration and invasion [47,48,49,50].

Another important mechanism involves NETs, a product of the severe inflammation triggered by COVID-19. Previous studies proposed that NETs may play a key role in the reawakening of dormant cancer cells [13,51,52]. Indeed, a landmark study by Albrengues et al. (2018) experimentally proved that proteases contained in NETs cleave laminin in the extracellular matrix, thereby activating integrin signaling in dormant cancer cells and prompting them to re-enter the cell cycle [18]. Our previous work, which showed increased neutrophil and NET formation signaling in the PBMC transcriptome of severe COVID-19 patients, is strong evidence that this mechanism may be active in our cohort as well [19].

The oncogenic potential of the virus may also be related to more direct mechanisms beyond the host’s inflammatory response. An in vitro study demonstrated that live SARS-CoV-2 virus directly increased the proliferation of ACE2 receptor-expressing prostate cancer cells and the expression of proliferation markers like Ki-67 [51]. This suggests that the viral infection itself can shift the biology of cancer cells in a pro-tumorigenic direction, independent of host inflammation. Moreover, SARS-CoV-2 may have indirect oncogenic effects. Chen et al. found that SARS-CoV-2 structural proteins can trigger the lytic reactivation of a known oncogenic virus, Kaposi’s sarcoma-associated herpesvirus (KSHV) [17]. This raises the possibility that COVID-19 could indirectly increase cancer risk by activating other latent oncogenic viruses in the host. The transcriptomic results of Salgado-Albarrán et al. showed that SARS-CoV-2 infection profoundly alters the epigenetic landscape of the host cell, involving transcription factors like JUN and epigenetic factors like EP300 [53]. Our findings strongly support this epigenetic reprogramming hypothesis. The persistent activation of the transcription factors JUN (a component of AP-1) and EGR1 (an Early Growth Response factor) suggests that the inflammatory signal is converted into a permanent pro-proliferative command, contributing to the “sustaining proliferative signaling” hallmark [54,55,56,57,58,59,60,61]. Moreover, the long-term regulation of Histone H3 variants, H3F3C and HIST2H3D, indicates an altered chromatin structure in PBMCs, which may lead to the persistent accessibility of pro-inflammatory and pro-proliferative genes [62,63,64,65,66]. This persistent epigenetic change supports the formation of the systemic “inflammatory memory” mentioned by Qian et al., setting the stage for an accelerated response to future oncogenic signals. We continue to follow the patients to determine whether the transcriptomic findings of our study reflect cancer development in real life. Among the study participants, one patient under follow-up developed thymoma and metastatic colorectal cancer, and another patient was diagnosed with a neuroendocrine tumor. These malignancies were observed within the 4-year follow-up period; however, the long-term outcomes of these patients are still being monitored (Supplementary Table S1).

In conclusion, this study provides robust transcriptomic evidence that severe COVID-19 infection leaves a permanent, systemic, and pro-oncogenic immunological scar that persists long after infection and can increase cancer risk. The identification of specific molecular targets like SQSTM1/P62 and the enrichment of cancer-related pathways elucidate the biology underlying this process. Our findings, combined with the powerful evidence from both clinical observations and large human cohorts as well as experimental models, definitively underscore the critical importance of long-term oncological surveillance for patients who have recovered from severe COVID-19 for potential malignancy development or recurrence. This must be a major priority area for healthcare systems in the post-pandemic era.

## Data Availability

Data are contained within the article and Supplementary Materials. The raw data have been deposited in NCBI Short Read Archive (SRA, https://www.ncbi.nlm.nih.gov/sra, accessed on 19 April 2024) under BioProject PRJNA895325.

## Supplementary Materials

The following supporting information can be downloaded at https://www.mdpi.com/article/10.3390/v17121608/s1: Table S1: Clinical descriptive data for two cases.

## References

[1] Crook H., Raza S., Nowell J., Young M., Edison P. (2021). Long COVID—Mechanisms, risk factors, and management. BMJ.

[2] Shah W., Hillman T., Playford E.D., Hishmeh L. (2021). Managing the long term effects of COVID-19: Summary of NICE, SIGN, and RCGP rapid guideline. BMJ.

[3] Antoniou K.M., Vasarmidi E., Russell A.M., Andrejak C., Crestani B., Delcroix M., Dinh-Xuan A.T., Poletti V., Sverzellati N., Vitacca M. (2022). European Respiratory Society statement on long COVID follow-up. Eur. Respir. J..

[4] Tian J., Yuan X., Xiao J., Zhong Q., Yang C., Liu B., Cai Y., Lu Z., Wang J., Wang Y. (2020). Clinical characteristics and risk 17 factors associated with COVID-19 disease severity in patients with cancer in Wuhan, China: A multicentre, retrospective, cohort study. Lancet Oncol..

[5] Zhang L., Zhu F., Xie L., Wang C., Wang J., Chen R., Jia P., Guan H.Q., Peng L., Chen Y. (2020). Clinical characteristics of COVID21 19-infected cancer patients: A retrospective case study in three hospitals within Wuhan, China. Ann. Oncol..

[6] Rogado J., Pangua C., Serrano-Montero G., Obispo B., Marino A.M., Pérez-Pérez M., Lara M.Á. (2020). COVID-19 and lung cancer: A greater fatality rate?. Lung Cancer.

[7] Curigliano G. (2020). Cancer patients and risk of mortality for COVID-19. Cancer Cell.

[8] Giannakoulis V.G., Papoutsi E., Siempos I.I. (2020). Effect of Cancer on Clinical Outcomes of 14 Patients With COVID-19: A Meta-Analysis of Patient Data. JCO Glob. Oncol..

[9] Liu G.E., Cai W., Liu H., Jiang H.H., Wang H. (2021). Malignancy is a risk factor for higher COVID-19 severity: A meta-analysis. Turk. J. Med. Sci..

[10] Monroy-Iglesias M.J., Tremble K., Russell B., Moss C., Dolly S., Sita-Lumsden A., Cortellini A., Pinato D.J., Rigg A., Karagiannis S.N. (2022). Long-term effects of COVID-19 on cancer patients: The experience from Guy’s Cancer Centre. Future Oncol..

[11] Tutuncuoglu B., Cakir M., Batra J., Bouhaddou M., Eckhardt M., Gordon D.E., Krogan N.J. (2020). The Landscape of Human Cancer Proteins Targeted by SARS-CoV-2. Cancer Discov..

[12] Au L., Boos L.A., Swerdlow A., Byrne F., Shepherd S.T.C., Fendler A., Turajlic S., CAPTURE investigators (2020). Cancer, COVID-19, and Antiviral Immunity: The CAPTURE Study. Cell.

[13] Francescangeli F., De Angelis M.L., Zeuner A. (2020). COVID-19: A potential driver of immune-mediated breast cancer recurrence?. Breast Cancer Res..

[14] Saygideger Y., Sezan A., Candevir A., Demir B.S., Güzel E., Baydar O., Derinoz E., Komur S., Kuscu F., Ozyılmaz E. (2021). COVID-19 patients’ sera induce epithelial mesenchymal transition in cancer cells. Cancer Treat. Res. Commun..

[15] Ager E.I., Neo J., Christophi C. (2008). The renin-angiotensin system and malignancy. Carcinogenesis.

[16] Bhardwaj K., Liu P., Leibowitz J.L., Kao C.C. (2012). The coronavirus endoribonuclease Nsp15 interacts with retinoblastoma tumor suppressor protein. J. Virol..

[17] Chen J., Dai L., Barrett L., James J., Plaisance-Bonstaff K., Post S.R., Qin Z. (2021). SARS-CoV-2 proteins and anti-COVID-19 drugs induce lytic reactivation of an oncogenic virus. Commun. Biol..

[18] Albrengues J., Shields M.A., Ng D., Park C.G., Ambrico A., Poindexter M.E., Upadhyay P., Uyeminami D.L., Pommier A., Küttner V. (2018). Neutrophil extracellular traps produced during inflammation awaken dormant cancer cells in mice. Science.

[19] Kayalar O., Cetinkaya P.D., Eldem V., Argun Baris S., Kokturk N., Kuralay S.C., Rajabi H., Konyalilar N., Mortazavi D., Korkunc S.K. (2024). Comparative Transcriptomic Analyses of Peripheral Blood Mononuclear Cells of COVID-19 Patients without Pneumonia and with Severe Pneumonia in the First Year of Follow-Up. Viruses.

[20] Izad M., Ahmadi E., Changaei M., Teymouri A., Alipour B. (2023). Cancer Related-Genes Enriched in Peripheral Blood Mononuclear Cells (PBMCs) of COVID-19 Patients. A Bioinformatics Study. Glob. J. Life Sci. Biol. Res..

[21] Baris S.A., Toprak O.B., Cetinkaya P.D., Fakili F., Kokturk N., Kul S., Kayalar O., Tutuncu Y., Azak E., Kuluozturk M. (2022). The predictors of long–COVID in the cohort of Turkish Thoracic Society–TURCOVID multicenter registry: One year follow–up results. Asian Pac. J. Trop. Med..

[22] Kokturk N., Babayigit C., Kul S., Cetinkaya P.D., Nayci S.A., Baris S.A., Karcioglu O., Aysert P., Irmak I., Yuksel A.A. (2021). The predictors of COVID-19 mortality in a nationwide cohort of Turkish patients. Respir. Med..

[23] Ewels P., Magnusson M., Lundin S., Käller M. (2016). MultiQC: Summarize analysis results for multiple tools and samples in a single report. Bioinformatics.

[24] Chen S., Zhou Y., Chen Y., Gu J. (2018). fastp: An ultra-fast all-in-one FASTQ preprocessor. Bioinformatics.

[25] Shen W., Le S., Li Y., Hu F. (2016). SeqKit: A Cross-Platform and Ultrafast Toolkit for FASTA/Q File Manipulation. PLoS ONE.

[26] Kim D., Paggi J.M., Park C., Bennett C., Salzberg S.L. (2019). Graph-based genome alignment and genotyping with HISAT2 and HISAT-genotype. Nat. Biotechnol..

[27] Tarasov A., Vilella A.J., Cuppen E., Nijman I.J., Prins P. (2015). Sambamba: Fast processing of NGS alignment formats. Bioinformatics.

[28] Liao Y., Smyth G.K., Shi W. (2014). featureCounts: An efficient general purpose program for assigning sequence reads to genomic features. Bioinformatics.

[29] Love M.I., Huber W., Anders S. (2014). Moderated estimation of fold change and dispersion for RNA-seq data with DESeq2. Genome Biol..

[30] Xia J., Gill E.E., Hancock R.E. (2015). NetworkAnalyst for statistical, visual and network-based meta-analysis of gene expression data. Nat. Protoc..

[31] Szklarczyk D., Gable A.L., Nastou K.C., Lyon D., Kirsch R., Pyysalo S., Doncheva N.T., Legeay M., Fang T., Bork P. (2021). The STRING database in 2021: Customizable protein-protein networks, and functional characterization of user-uploaded gene/measurement sets. Nucleic Acids Res..

[32] Suehnholz S.P., Nissan M.H., Zhang H., Kundra R., Nandakumar S., Lu C., Carrero S., Dhaneshwar A., Fernandez N., Xu B.W. (2024). Quantifying the Expanding Landscape of Clinical Actionability for Patients with Cancer. Cancer Discov..

[33] Chakravarty D., Gao J., Phillips S.M., Phillips S., Kundra R., Zhang H., Wang J., Rudolph J.E., Yaeger R., Soumerai T. (2017). OncoKB: A Precision Oncology Knowledge Base. JCO Precis. Oncol..

[34] Menyhart O., Kothalawala W.J., Gyorffy B. (2024). A gene set enrichment analysis for the cancer hallmarks. J. Pharm. Anal..

[35] Li T., Fu J., Zeng Z., Cohen D., Li J., Chen Q., Li B., Liu X.S. (2020). TIMER2.0 for analysis of tumor-infiltrating immune cells. Nucleic Acids Res..

[36] Győrffy B. (2024). Integrated analysis of public datasets for the discovery and validation of survival-associated genes in solid tumors. Innovation.

[37] Chia S.B., Johnson B.J., Hu J., Vermeulen R., Chadeau-Hyam M., Guntoro F., Montgomery H., Boorgula M., Sreekanth V., Goodspeed A. (2024). Respiratory viral infection promotes the awakening and outgrowth of dormant metastatic breast cancer cells in lungs. Res. Sq..

[38] Qian W., Wei X., Barros A.J., Ye X., Yu Q., Young S.P., Yeatts E.V., Park Y., Li C., Almeida-Santos G. (2025). Respiratory viral infections prime accelerated lung cancer growth. bioRxiv.

[39] Kopan R., Ilagan M.X.G. (2009). The canonical Notch signaling pathway: Unfolding the activation mechanism. Cell.

[40] Shah P.A., Huang C., Li Q., Kazi S.A., Byers L.A., Wang J., Johnson F.M., Frederick M.J. (2020). NOTCH1 Signaling in Head and Neck Squamous Cell Carcinoma. Cells.

[41] Li B., Huang L., Ruan J. (2024). PKMYT1 Promotes Epithelial-Mesenchymal Transition Process in Triple-Negative Breast Cancer by Activating Notch Signaling. Rev. Investig. Clin..

[42] Ntziachristos P., Lim J.S., Sage J., Aifantis I. (2014). From Fly Wings to Targeted Cancer Therapies: A Centennial for Notch Signaling. Cancer Cell.

[43] Quillard T., Devallière J., Coupel S., Charreau B. (2010). Inflammation dysregulates notch signaling in endothelial cells: Implication of Notch2 and Notch4 to endothelial dysfunction. Biochem. Pharmacol..

[44] Wongchana W., Palaga T. (2012). Direct regulation of interleukin-6 expression by notch signaling in macrophages. Cell. Mol. Immunol..

[45] Roger L., Tomas F., Gire V. (2021). Mechanisms and Regulation of Cellular Senescence. Int. J. Mol. Sci..

[46] Hoare M., Ito Y., Kang T.W., Weekes M.P., Matheson N.J., Patten D.A., Shetty S., Parry A.J., Menon S., Salama R. (2016). NOTCH1 mediates a switch between two distinct secretomes during senescence. Nat. Cell Biol..

[47] Paunovic V., Vucicevic L., Misirkic Marjanovic M., Perovic V., Ristic B., Bosnjak M., Mandic M., Stevanovic D., Harhaji-Trajkovic L., Lalosevic J. (2023). Autophagy Receptor p62 Regulates SARS-CoV-2-Induced Inflammation in COVID-19. Cells.

[48] Koepke L., Hirschenberger M., Hayn M., Kirchhoff F., Sparrer K.M. (2021). Manipulation of autophagy by SARS-CoV-2 proteins. Autophagy.

[49] Mukherjee S., Maddalena M., Lü Y., Martinez S., Nataraj N.B., Noronha A., Sinha S., Teng K., Cohen-Kaplan V., Ziv T. (2022). Cross-talk between mutant p53 and p62/SQSTM1 augments cancer cell migration by promoting the degradation of cell adhesion proteins. Proc. Natl. Acad. Sci. USA.

[50] Smith A.G., Macleod K.F. (2019). Autophagy, cancer stem cells and drug resistance. J. Pathol..

[51] Serwaa A., Oyawoye F., Owusu I.A., Dosoo D., Manu A.A., Sobo A.K., Fosu K., Olwal C.O., Quashie P.K., Aikins A.R. (2024). In vitro analysis suggests that SARS-CoV-2 infection differentially modulates cancer-like phenotypes and cytokine expression in colorectal and prostate cancer cells. Sci. Rep..

[52] Francescangeli F., De Angelis M.L., Baiocchi M., Rossi R., Biffoni M., Zeuner A. (2020). COVID-19–Induced Modifications in the Tumor Microenvironment: Do They Affect Cancer Reawakening and Metastatic Relapse?. Front. Oncol..

[53] Salgado-Albarrán M., Navarro-Delgado E.I., Del Moral-Morales A., Alcaraz N., Baumbach J., González-Barrios R., Soto-Reyes E. (2021). Comparative transcriptome analysis reveals key epigenetic targets in SARS-CoV-2 infection. npj Syst. Biol. Appl..

[54] Eferl R., Wagner E.F. (2003). AP-1: A double-edged sword in tumorigenesis. Nat. Rev. Cancer.

[55] Cheong J.G., Ravishankar A., Sharma S., Parkhurst C.N., Grassmann S.A., Wingert C.K., Laurent P., Ma S., Paddock L., Miranda I.C. (2023). Epigenetic memory of coronavirus infection in innate immune cells and their progenitors. Cell.

[56] Papavassiliou A.G., Musti A.M. (2020). The Multifaceted Output of c-Jun Biological Activity: Focus at the Junction of CD8 T Cell Activation and Exhaustion. Cells.

[57] Nair P., Muthukkumar S., Sells S.F., Han S.S., Sukhatme V.P., Rangnekar V.M. (1997). Early growth response-1-dependent apoptosis is mediated by p53. J. Biol. Chem..

[58] Virolle T., Adamson E.D., Baron V., Birle D., Mercola D., Mustelin T., de Belle I. (2001). The Egr-1 transcription factor directly activates PTEN during irradiation-induced signalling. Nat. Cell Biol..

[59] Wang B., Guo H., Yu H., Chen Y., Xu H., Zhao G. (2021). The role of the transcription factor EGR1 in cancer. Front. Oncol..

[60] Zou K., Zeng Z. (2023). Role of early growth response 1 in inflammation-associated lung diseases. Am. J. Physiol. Lung Cell. Mol. Physiol..

[61] Yuan L., Fung T.S., He J., Chen R.A., Liu D.X. (2022). Modulation of viral replication, apoptosis and antiviral response by induction and mutual regulation of EGR and AP-1 family genes during coronavirus infection. Emerg. Microbes Infect..

[62] Okur V., Chen Z., Vossaert L., Peacock S., Rosenfeld J., Zhao L., Du H., Calamaro E., Gerard A., Zhao S. (2021). De novo variants in H3-3A and H3-3B are associated with neurodevelopmental delay, dysmorphic features, and structural brain abnormalities. npj Genom. Med..

[63] Marzluff W.F., Gongidi P., Woods K.R., Jin J., Maltais L.J. (2002). The human and mouse replication-dependent histone genes. Genomics.

[64] Pereira E.P.V., da Silva Felipe S.M., de Freitas R.M., da Cruz Freire J.E., Oliveira A.E.R., Canabrava N., Soares P.M., van Tilburg M.F., Guedes M.I.F., Grueter C.E. (2023). Transcriptional Profiling of SARS-CoV-2-Infected Calu-3 Cells Reveals Immune-Related Signaling Pathways. Pathogens.

[65] Yıldırım Ç., Yay F., İmre A., Soysal O., Yıldırım H.Ç. (2024). CXCL10, SCGN, and H2BC5 as Potential Key Genes Regulated by HCV Infection. Genes.

[66] Kee J., Thudium S., Renner D.M., Glastad K., Palozola K., Zhang Z., Li Y., Lan Y., Cesare J., Poleshko A. (2022). SARS-CoV-2 disrupts host epigenetic regulation via histone mimicry. Nature.

