# Supplementary material for: Long-Term Transcriptomic Reprogramming in Peripheral Blood Mononuclear Cells of Severe COVID-19 Survivors Reveals Pro-Oncogenic Signatures and Cancer-Associated Hub Genes
Source: Viruses. 2025 Dec 12;17(12):1608. doi: 10.3390/v17121608 (PMC12737505; doi:10.3390/v17121608)
Supplement: Supplementary file 1 [file viruses-17-01608-s001.zip › viruses-4005524-supplementary.pdf]

SUPPLEMENTARY MATERIAL:

Table S1: Clinical descriptive data for two cases.

	Age	Sex	COVID19+	Pneumonia	The time of contracting COVID-19	Time of PBMC sampling	Type of cancer	Time of cancer diagnosis	Alive or dead?
Case 1	66	Male	Positive	Severe-Pneumonia Group	April 2020	2021	Thymoma<br><br>Metastatic Colon Cancer	October 2020<br><br>May 2024	Alive
Case 2	50	Male	Positive	Severe-Pneumonia Group	April 2020	2021	Gastric Neuroendocrine tumor	August 2020	Alive
